# Effects of *Origanum majorana* on Breast Cancer Cells: An Alternative to Chemotherapy?

**DOI:** 10.3390/metabo13101083

**Published:** 2023-10-16

**Authors:** Zoe Sanders, Bridgette A. Moffitt, Madeleine Treaster, Ashley Larkins, Nicholas Khulordava, Jennifer Benjock, Jillian Spencer, Krista Henrie, Matthew J. Wurst, Abigail Broom, Noah Tamez, Gianna DeRosa, McKenzie Campbell, Elizabeth Keller, Addison Powell, Donna Weinbrenner, Ludovico Abenavoli, W. Jeffery Edenfield, Ki Chung, Luigi Boccuto, Diana Ivankovic

**Affiliations:** 1 Departments of Biology, Center for Cancer Research, Anderson University, 316 Boulevard, Anderson, SC 29621, USA; sandersz@email.sc.edu (Z.S.); abroom100@andersonuniversity.edu (A.B.); apowell114@andersonuniversity.edu (A.P.); 2 Department of Healthcare Genetics, School of Nursing, Clemson University, Clemson, SC 29634, USAjspenc5@clemson.edu (J.S.); lboccut@clemson.edu (L.B.); 3 Department of Chemistry, Anderson University, Anderson, SC 29621, USA; 4 Department of Biological Sciences, Clemson University, Clemson, SC 29634, USA; 5 Department of Health Sciences, University “Magna Graecia”, Viale Europa—Germaneto, 88100 Catanzaro, Italy; l.abenavoli@unicz.it; 6 Prisma Health Cancer Institute, Greenville, SC 29605, USAki.chung@prismahealth.org (K.C.); 7 Department of Biological Sciences, Anderson University, 316 Boulevard, Anderson, SC 29621, USA

**Keywords:** breast cancer, marjoram, cancer treatment, cancer metabolism, MCF-7

## Abstract

Recent studies have reported several beneficial effects of natural compounds on cancerous cells, highlighting their use for future treatments. These preliminary findings have encouraged experiments with natural substances, such as plant extracts, to examine both cytotoxic and mitogenic effects and find alternative treatments for diseases such as breast cancer. This study examines the effects of microwave-assisted and ethanol maceration of marjoram (*Origanum majorana*) on MCF-7 breast cancer cell lines and normal breast tissue cell lines used as controls. Marjoram extracts displayed a cytotoxic effect on the MCF-7 cell lines and a mitogenic effect on the control cell lines at the MTS test. The metabolic profiles of MCF-7 and control cell lines were also assessed using the Biolog Phenotype Mammalian Metabolic (PM-M) platform and revealed statistically significant differences in the utilization of energy sources, metabolic activity in the presence of certain ionic species, and responses to metabolic effectors, such as stimulant/catabolic compounds and steroid hormones. Exposure to marjoram extracts exerted positive effects on the MCF-7 cells on the abnormal utilization of energy sources and the responses to metabolic effectors, while no major effects were detected on control cells. These effects were compared to the metabolic impact of the chemotherapeutic agent doxorubicin, which showed profound cytotoxic effects on both cancerous and normal breast cells. In conclusion, our in vitro evidence indicates that marjoram extracts are a promising alternative to chemotherapy in breast cancer since they can successfully eliminate cancerous cells by affecting their metabolic capacity to proliferate without inducing noticeable adverse effects on normal breast tissue.

## 1. Introduction

Treatment of breast cancer has achieved remarkable successes in the recent decade: the combination of early diagnosis, genetic characterization, and surgical, chemotherapeutic, and radiotherapeutic approaches has dramatically increased the survival rate of various types of breast cancers, particularly those in the early stages. Among the most commonly employed drugs in chemotherapeutic protocols, doxorubicin (adriamycin) has shown promising results, as well as severe adverse effects, mostly related to cardiotoxicity [1].

The search for alternative and less toxic therapies for treating breast cancer has recently led to the development of new approaches based on natural compounds. Natural compounds such as curcumin, biochanin A (red clover), and genistein (soy products) have been shown to have potent cytotoxic effects on breast cancer cell lines, with the ability to induce apoptosis, regulate gene expression, and down-regulate proliferation signaling [2]. These results highlight the potential for the development of effective anti-cancer treatments using non-synthetic compounds. One genus of plants shown to exhibit antioxidant properties and the potential to combat cancer is the *Origanum* genus. These plants are classified as members of the Lamiaceae (mint) family, encompassing more than 200 genera [3]. The *Origanum* genus comprises a diverse range of plants, including 44 species, 6 subspecies, 3 botanical varieties, and 18 hybrid forms [3]. Though many of the species within the genus thrive in the Mediterranean, their cultivation has extended all over the world [4]. *Origanum majorana*, more commonly referred to as sweet marjoram [5], has historically been valued for its therapeutic, culinary, aromatic, and ornamental uses. Initially used as an antiseptic, marjoram was integrated into ancient Greece as a symbol of love and honor and in ancient Egypt as both a disinfectant and food preservative [5]. *O. majorana* is characterized for its robust, spicy flavor and pleasant aroma, making it a favored addition to a variety of food preparations [5]. As a medicinal herb, *O. majorana* has traditionally been used as an anti-inflammatory agent with anti-pyretic properties and has been shown to be effective against allergies, fevers, influenza, gastrointestinal disorders, and hypertension [5,6,7]. Furthermore, *O. majorana* has demonstrated utility in the management of respiratory infections, diabetes, and menstrual pain [7].

Several compounds in *O. majorana* have been proven to have multiple beneficial effects on human metabolism: both in vitro and in vivo experiments have confirmed anti-diabetic effects of marjoram extracts, such as reduction in fasting glucose, lower glycated hemoglobin, improved oral glucose tolerance test results, and greater hepatic glycogen compared to the diabetic control [8]. Marjoram extract also exerts anti-inflammatory effects by reducing the levels of iNOS expressed by macrophages after stimulation and preventing NO production [9]; and a reduction in levels of cytokines such as IL-1β, IL-6, and TNF-α has also been detected [10].

Anti-cancer effects of *O. majorana* ethanol extract have been described in various human cancer cell lines and are based on the suppression of colony growth, cell viability, and induction of mitotic arrest, DNA damage, triggering of the abortive autophagy, and activation of a caspase-3- and 7-dependent extrinsic apoptotic pathway [11,12,13]. Genes participating in fatty acid and cholesterol biosynthesis such as *acetyl CoA synthase* (*ACC*), *3-hydroxy-3-methylglutaryl-coenzyme A reductase* (*HMGCR*), *sterol regulatory element-binding protein 1* (*SPREBP1*), and *fatty acid synthase* (*FASN*) were found to be reduced in transcript profile and protein accumulation resulted in cell growth inhibition. Based on GCMS experiments and analysis, terpinen-4-ol and cis-sabinene hydrate are the two main components of marjoram. Specifically, the antibacterial and anti-inflammatory properties of marjoram can be attributed to the sabinene hydrate found in the plant [14]. Most notably, volatile oils from *Origanum syriacum* and *Origanum vulgare* (within the same family as marjoram) cultures have been shown to produce a statistically significant decrease in tumor cell proliferation in MCF-7 cell lines [15].

Due to its antiproliferative and apoptotic effects on various cell lines, sabinene hydrate and the other various components of marjoram are being studied extensively and applied to cancer therapies. In this experiment, we monitor the effects of extracts from dried marjoram plants and pills on MCF-7 and MCF-12/MCF-12F cell lines to test the hypothesis that this plant and its components promote antiproliferative and apoptotic properties.

## 2. Materials and Methods

### 2.1. Cell Nutrition and Maintenance

MCF-7 cells were grown in T-75 cell culture flasks in an incubator at 37 °C with flowing CO_2_ at 5% concentration within the incubator. The MCF-7 cells were maintained with DMEM complete growth medium. The complete growth medium consisted of 500 mL of DMEM growth medium, 50 mL of fetal bovine serum (FBS) (15070063, VWR International, LLC, Suwannee, GA, USA), 500 μL of 10,000X HEPES (15630106, Thermo Fisher Scientific, Greenville, SC, USA), and 5 mL of penicillin–streptomycin (15070063, Thermo Fisher Scientific, Greenville, SC, USA). Following the addition of all components to the original 500 mL of DMEM medium, the mixture was vacuum-filtered (0.1 µm) to ensure sterile nutrients for the cells. The complete growth medium was replaced every 48 h to ensure that the cells remained properly nourished. To achieve this, the old medium was removed, and a fresh 15 mL of complete growth medium was added to the culture flasks. When the cell culture was between 60–75% confluency, the cells were split. The first step in this process was removing the old growth medium from the flask. Following this, 25 mL of phosphate-buffered saline (PBS) (SH30256LS, Thermo Fisher Scientific, Greenville, SC, USA) was added to the culture flask to wash the cells. Once the PBS was in the culture flask, the flask was gently slid back and forth 80 times to ensure the cells were thoroughly washed, and then the PBS was removed. Subsequently, 7.5 mL of trypsin (25200056, Thermo Fisher Scientific, Greenville, SC, USA) was added to the culture flask. Trypsin was added to unbind the cells from each other and away from the culture flask. While the trypsin was inside the culture flask, it was gently shaken back and forth 80 times. After being placed in the incubator for 5 min, the solution was centrifuged for 5 min at 800 rpm. The supernatant was discarded, and the new pellet of cells was resuspended in 15 mL of cell growth media. This was then aliquoted to new flasks in a 1:5 dilution, using 3 mL of the cell and media solution, as well as 12 mL of fresh media in each flask.

### 2.2. Extraction Procedures

#### 2.2.1. Microwave-assisted Extraction

The ETHOSTM X, microwave oven from Milestone Inc. (Sorisole, Italy) was used, with an infrared temperature probe, and a max power of 1600 W. The extraction was carried out in ethanol, at the max temperature of 120 °C for ten minutes, and under pressure, following the EPA Method 3546 [16]. Plant material and ethanol were added to the cylindrical glass tubes (about 20–30 mL). The glass tubes were then placed in the plastic sleeves of the vessels and the tops of the vessels were tightly screwed on, before placing them symmetrically in the microwave oven rotor. The samples were kept for ten minutes at 120 °C at the max power of 1600 W. After the heating period was completed, vessels were kept closed for at least 45 min to allow for cooling before they were opened. The solvent was decanted and removed by rotary evaporation and the plant’s residue was weighed and stored in the refrigerator at 4 °C until further use.

#### 2.2.2. Ethanol Maceration

To prepare the marjoram extraction in ethanol, 4 g of powder obtained from marjoram pills was added to 50 mL of 100% ethanol. After macerating for 24 h, the mixture was decanted and then added to a 100 mL round-bottomed flask. The ethanol was then removed using rotary evaporation and the remaining residue was separated into 0.1 g amounts and used to prepare stock solutions.

### 2.3. Preparation of the Extract’s Solutions in the Complete Media

Extract preparation of *Origanum majorana* began by weighing 0.1 g of the dried extract (either soxhlet or microwave-assisted) and placing the extract into a 50 mL centrifuge tube. Complete medium for the corresponding cell line was added to bring the final volume to 10 mL. Until used, all extracts are stored in the refrigerator at 4 °C.

### 2.4. Plate Preparation

Before plating, the MCF-7 cell line was first split and divided based on a concentration of 1.0 × 10⁵ cells/mL, using a hemocytometer for counting. First, the medium was removed from one of the T-75 culture flasks that contained the MCF-7 cells. Phosphate-buffered saline (PBS) (5 mL) was added to the flask to remove any protective agents from the cells and the T-75 tissue culture flask was gently slid back and forth to rinse the cells. PBS was removed, and 15 mL of trypsin was added for detachment purposes. Once again, the T-75 tissue culture flask was delicately moved back and forth and then incubated for 5 min at 37 °C. Following incubation, trypsin was immediately removed, and fresh medium (25 mL) was added to each of the T-75 culture flasks to remove the attached cells. The same procedure was repeated for each of the flasks.

To obtain a cell count, 20 µL of medium/cell solution was removed and placed into a micro-centrifuge tube. The number of living cells was then calculated using an automated counter and the concentration of the cell suspension was adjusted for 1.0 × 10⁵ cells/mL. The cultured MCF-7 cells were aliquoted to distribute 100 µL in each well for columns 3–11 in a 96-well plate, and incubated for 48 h at 37 °C, at 5% CO_2_. Following the 48 h incubation period, the wells were all emptied onto a sterilized paper towel. All wells were washed with 100 μL of PBS and then decanted, followed by treatments with extract mixture concentrations (100 µL) into each appropriate well. Beginning with column one; this column contained only medium (M). Column two contained medium and extract only (ME). Columns one and two were negative controls, which ensured no contamination in the medium or extract and the reliability of results. Column three contained MCF-7 cells with medium and hydrogen peroxide (H_2_O_2_) at 3% in a 1:1 ratio (MCH_2_O_2_). Column three was a positive control, resulting in cell death. Columns 4–10 contained cells along with the different marjoram extract concentrations. Columns 4–10 contained cells and extract in concentrations of 1000 μg/mL, 500 μg/mL, 100 μg/mL, 50 μg/mL, 10 μg/mL, 5 μg/mL, and 1 μg/mL, respectively. Column 11 was another negative control, with only cells and complete growth medium, to prove that the cells were healthy with high proliferation.

Following 48 h of incubation, the plates were tested with an MTS (3-(4,5-dimethylthiazol-2-yl)-5-(3-carboxymethoxyphenyl)-2-(4-sulfophenyl)-2H-tetrazolium) assay to measure cell viability. A reservoir was filled with fresh medium and MTS solution (CellTiter 96^®^ AQueous One Solution Cell Proliferation Assay (MTS), Promega, Madison, WI, USA), and mixed well under light-sensitive conditions. After the 96-well plates were taken out of the incubator, the plates were decanted onto a paper towel and each well was washed with 100 µL of PBS. The MTS mixture (120 µL) was added to wells in columns 1–11 using a multi-channel pipette. Column three served as a positive control, containing cells, MTS solution, and hydrogen peroxide at 3% in a 1:1 ratio. The plate was then incubated for 30 min at 37 °C, at 5% CO_2_. The absorbance of each plate was measured on an ELISA plate reader at 30 min intervals, for a total of 2 h at 450 nm.

### 2.5. Biolog Metabolic Arrays

The Phenotype Mammalian MicroArray (PM-M) developed by Biolog (Hayward, CA, USA) is designed to measure the cellular production of nicotinamide adenine dinucleotide, reduced form, (NADH) in the presence of different compounds. The methodology employs four microplates with diverse compounds utilized as energy sources, including carbon energy sources (plate PM-M1), as well as amino acids, both alone and as dipeptides (plates PM-M2 to M4). Another four microplates contain metabolic effectors, including ions (plate PM-M5), hormones, cytokines, and growth factors (plates PM-M6 to M8). Each well contains a single chemical and the production of NADH per well is monitored using a colorimetric redox dye chemistry [17]. PM-M plates were incubated with 20,000 cells per well in a volume of 50 μL, using the modified Biolog IF-M1 medium. This medium was prepared for plates PM-M1 to M4 by adding the following to 100 mL of Biolog IF-M1: 1.1 mL of 100× penicillin/streptomycin solution, 0.16 mL of 200 mM Glutamine (final concentration 0.3 mM), and 5.3 mL of fetal bovine serum (final concentration 5%). For plates PM-M5 to M8, the fetal bovine serum was replaced by 5.5 mL of 100 mM glucose (final concentration 5%), which was used as the sole energy source. The cells were incubated for 48 h at 37 °C in 5% CO_2_.

For the experiments with candidate compounds, 10 μL of 500 μg/mL marjoram extracts or 10 μL of 0.5 μM doxorubicin were added to the cell suspension on day 1. During the 48 h incubation, the only energy source available to the cells was the chemical in the well. After this first incubation, Biolog Redox Dye Mix MB was added (10 μL/well) and the plates were incubated under the same conditions for an additional 24 h, during which time the cells metabolized the sole carbon source in the well. As the cells metabolize the energy source, tetrazolium dye in the media is reduced, producing a purple color according to the amount of NADH generated. All experiments were conducted in triplicates.

At the end of the 24 h incubation, the plates were analyzed utilizing a microplate reader with readings at 590 and 750 nm. The first value (A_590_) indicated the highest absorbance peak of the redox dye and the second value (A_750_) measured the background noise. The relative absorbance (A_590–750_) was calculated per well.

### 2.6. Statistical Analysis

Statistical analysis of Phenotype Mammalian MicroArray (PM-M) data was calculated via conducting Student’s *t*-tests comparing each test variable to the control, and separately, testing the two test variables against each other. We wanted to know how each group compares to another group specifically; the Student’s *t*-test was utilized with an α = 0.05.

ANOVA was not used because ANOVA is categorized as an omnibus type of test statistic, testing the explained variance of multiple groups of data for significant differences of several parameters simultaneously in a model. The ANOVA test is not able to identify the specific groups that were statistically significantly different from each other, simply that at least two of the groups were significantly different without identification.

When calculating results, α = 0.05 was considered as the threshold for significance. Using the Bonferroni adjustment to compensate for a type I error, we obtained α = 0.00227 for each graph and result.

## 3. Results

### 3.1. MTS Assay Results

**Figure 1:**
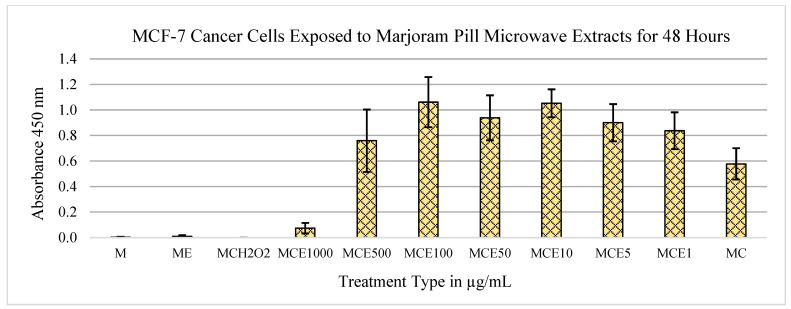
Effects of marjoram pills at an absorbance of 450 nm on MCF-7 cell line for 48 h. Where “M” is medium only, “E” is extract, “C” is cells, “H_2_O_2_” is hydrogen peroxide, and the following numbers correspond to the treatment type in μg/mL.

As shown in Figure 1, the marjoram extracts exert cytotoxic effects on the MCF-7 cells at the 1000 μg/mL concentration. A control of cells with medium alone was significantly different (*p* = 7.16 × 10^−6^). The data also show a statistically significant drop between the 500 μg/mL and 1000 μg/mL concentrations (*p* = 8.94 × 10^−5^). Thus, it can be concluded that between the concentrations of 500 and 1000 μg/mL, marjoram extracts have cytotoxic effects on MCF-7 breast cancer cells.

**Figure 2:**
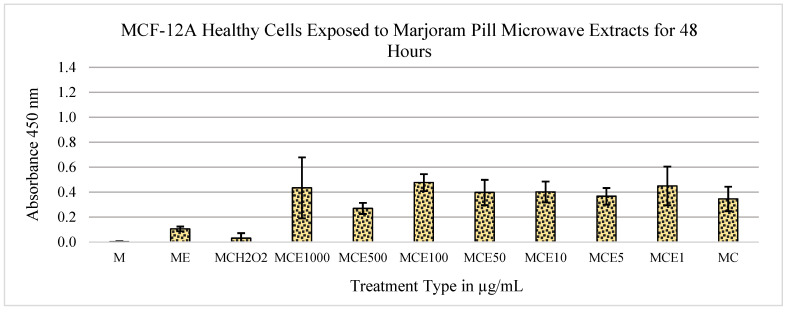
Effects of marjoram pills at an absorbance of 450 nm on MCF-12A cell line for 48 h. Where “M” is medium only, “E” is extract, “C” is cells, “H_2_O_2_” is hydrogen peroxide, and the following numbers correspond to the treatment type in μg/mL.

The other extract dilutions show no significant cytotoxic effects when compared to the control of medium and cells. Figure 2 shows no cytotoxic effects of the extract on the MCF-12A cell line.

**Figure 3:**
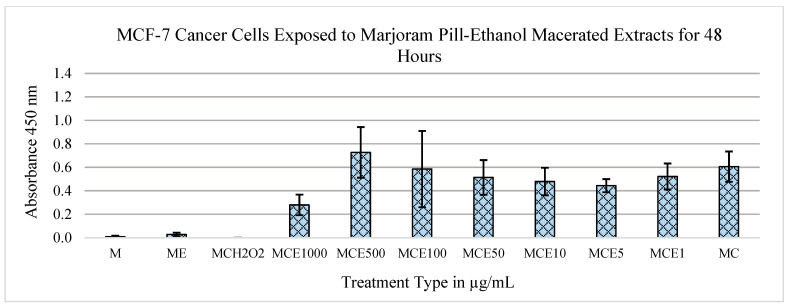
Effects of marjoram pills at an absorbance of 450 nm on MCF-7 cell line for 48 h (ethanol maceration). Where “M” is medium only, “E” is extract, “C” is cells, “H_2_O_2_” is hydrogen peroxide, and the following numbers correspond to the treatment type in μg/mL.

As seen in Figure 3, the MCF-7 cells treated with extracts of marjoram pills macerated in ethanol display a mitogenic trend up to concentrations of 500 μg/mL. At this concentration, extracts incorporating ethanol maceration display a statistically significant drop in cell concentration, indicating a trend of cytotoxic effects. A statistically significant reduction in live MCF-7 levels was detected between the concentrations of 500 μg/mL and 1000 μg/mL (*p* = 5.76 × 10^−4^). Based on this, the conclusion was drawn that between these concentrations, the MCF-7 cells had died. A statistically significant cytotoxic effect was also observed when we compared the data from the 1000 μg/mL concentration to the control of medium and cells (*p* = 3.41 × 10^−5^).

**Figure 4:**
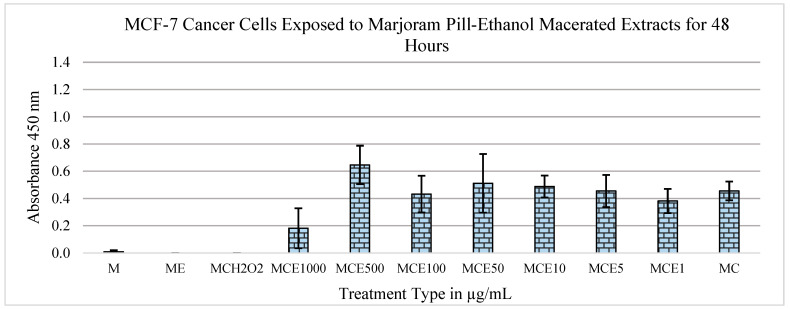
Effects of marjoram pills at an absorbance of 450 nm on MCF-7 cell line for 48 h (ethanol maceration). Where “M” is medium only, “E” is extract, “C” is cells, “H_2_O_2_” is hydrogen peroxide, and the following numbers correspond to the treatment type in μg/mL.

Figure 4 depicts the results of an independent replication of the findings shown in Figure 3. In this experiment, the concentrations of extract and control groups were kept the same. As seen in the figure, there is, once again, a mitogenic trend in the data between the 1 μg/mL and 500 μg/mL concentrations. Similarly to the previous findings (Figure 3), a statistically significant drop is observed in the concentration of MCF-7 cells between the 500 μg/mL and 1000 μg/mL concentrations, with a *p*-value of 2.57 × 10^−4^. When compared to the control group of medium and cells, the *p*-value was significant (0.0033; 0.00227).

**Figure 5:**
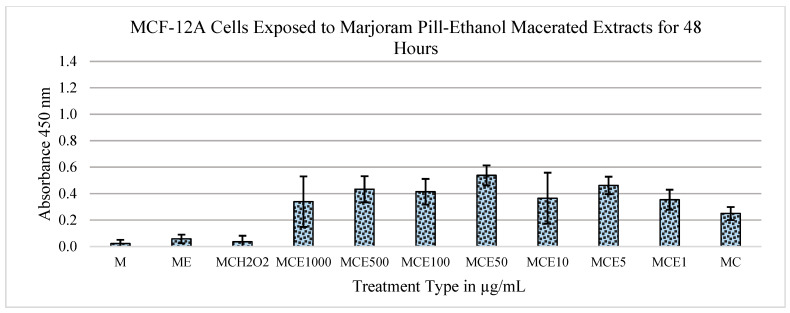
Effects of marjoram pills at an absorbance of 450 nm on MCF-12A cell line for 48 h (ethanol extraction). Where “M” is medium only, “E” is extract, “C” is cells, “H_2_O_2_” is hydrogen peroxide, and the following numbers correspond to the treatment type in μg/mL.

Figure 5 shows the effect of extracts from marjoram pills on the MCF-12A cell line: the overall trend shows the mitogenic effects of the extracts at each of the concentrations tested. When compared to the control of medium and cells, the *p*-values are almost statistically significant at the concentrations of 1000 μg/mL and 500 μg/mL, with *p*-values of 0.00547 and 0.0026, respectively. The data show no significant cytogenic effects or reduction in the concentration of MCF-12A cells, indicating that the cells remain alive after treatment with the marjoram extract.

### 3.2. Metabolic Results

#### 3.2.1. Baseline Metabolic Profile

A comparison of baseline metabolic profiles of MCF-7 versus control cell lines reveals significant differences in the utilization of energy sources (see Table S1 for the raw data). Data from the PM-M1 plate show an increased production of NADH in cancerous cells in the presence of dextrin, 3-methyl-glucose, α-methyl-d-glucoside, β-methyl-d-xyloside, and methyl-pyruvate (α < 0.05). These findings suggest a preferential use of methylated carbohydrates and increased utilization of anaerobic metabolic pathways, a phenomenon called the Warburg effect and largely described in cancerous cells due to their high replicating rate. A significant increase in NADH production (α < 0.05) in MCF-7 cells was also detected in wells containing alternative energy sources, such as sorbose, arabinose, and the nucleotides uridine and adenosine, suggesting compensatory mechanisms to support the cancerous cells with a high number of substrates, probably due to the lower efficacy of the anaerobic metabolism.

Plates PM-M2 to PM-M3 reveal significant differences in the metabolization of the amino acid tryptophan and a few dipeptides; however, the differences were not always coherent: in some cases, cancerous cells produced more energy than controls and in other cases, the opposite trend was noted, therefore, no consistent biological trend emerged from these data.

MCF-7 cells appeared to be less capable than controls in producing NADH in the presence of most of the ionic species contained in the PM-M5 plate: 35/96 wells (36.5%) showed a decreased energy production in cancer cells, as compared to MCF-12A/MCF-12F. The plate contains multiple wells for each ionic species to assess their effect at different concentrations. In our experiments, we noted that certain compounds induced a reduction in energy production in cancerous cells at more than one concentration, especially if they contained chloride, nitrite/nitrate, molybdate, tungstate, sodium, calcium, copper, cobalt, iron, or magnesium (see Table S1). Such findings suggest a reduced capacity of cancerous cells to adapt to metabolic distress, particularly to adjusting to extreme concentrations of certain ions and possibly changes in transmembrane gradients and pH.

Plates PM-M6, PM-M7, and PM-M8 contain hormones, cytokines, and other metabolic effectors at six increasing concentration data points each. As for PM-M5, the compounds are not utilized as energy sources, but the assays measure the capacity of the cells to produce NADH in the presence of glucose when exposed to these effectors. Increased metabolic responses in cancerous vs. control cells were detected in PM-M6 in the presence of 3-isobutyl-1-methylxanthine (4 concentration data points), epinephrine (4), creatine (1), progesterone (2), β-estradiol (1), 4,5-α-dihydrotestosterone (1), and aldosterone (2). These findings indicate a disrupted response to stimulant/catabolic compounds (methylxanthine and epinephrine) and steroid hormones (progesterone, estradiol, testosterone, and aldosterone).

Data from PM-M7 and PM-M8 plates show no significant differences between MCF-7 and control cells, except for increased NADH levels generated by cancerous cells when exposed to thyrotropin-releasing hormone and adenosine. In both cases, the differences were noted only at the highest concentration data point.

#### 3.2.2. Treatment with Candidate Compounds

After treatment with marjoram extracts, no major effects were detected on control cells (MCF-12A and MCF-12F) for all the PM-M plates, while the utilization of energy sources linked to glycolysis (PM-M1) by MCF-7 cells was reduced as compared to baseline, although some NADH levels were still significantly higher than the ones produced by the untreated controls (Table S1). Moreover, more alternative energy sources from PM-M1 were associated with increased NADH production by MCF-7 (α < 0.05), probably indicating that the reduced efficiency of the anaerobic glycolysis forced the cancerous cells to pursue different metabolic pathways further to produce the required levels of energy (Table S1). We also noticed a generalized increase in the utilization of amino acids and dipeptides by MCF-7 after exposure to marjoram, with most of the wells in plates PM-M2 to PM-M4 showing significantly higher levels of NADH as compared to untreated controls (α < 0.05): this trend confirms the switch to alternative and less efficient energy sources in cancerous cells.

Marjoram extracts fail to exert any relevant effect on the metabolic response to ions (PM-M5), suggesting that the phytochemicals in these extracts have no impact on the capacity of cancerous cells to adjust to changes in pH and/or transmembrane ionic gradients. Similarly, no effects of marjoram were detected in MCF-7 cells exposed to the compounds on plates PM-M7 and PM-M8 (Table S1).

**Figure 6:**
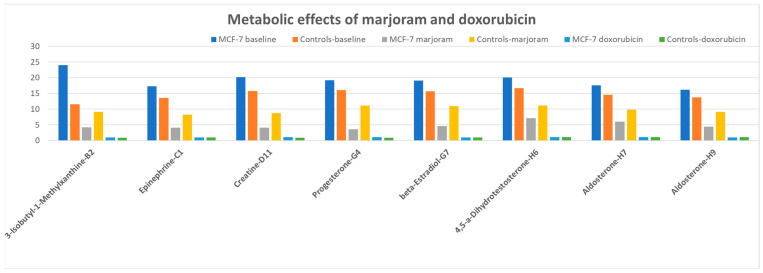
Differences in metabolic responses between MCF-7 and an average of MCF-12A and MCF-12F cell lines when exposed to compounds on the PM-M6 plate.

Interestingly, the most relevant effects exerted by marjoram were detected on the PM-M6 plate (Figure 6, Table S1): not only was the metabolic response by MCF-7 decreased and no longer significant when compared to untreated controls, but the lack of effects of marjoram on control cells produced an inversion in the ratio of metabolic responses with the treated MCF-7, producing lower levels of NADH than both treated and untreated controls when exposed to 3-isobutyl-1-methylxanthine, epinephrine, creatine, progesterone, β-estradiol, 4,5-α-dihydrotestosterone, and aldosterone.

Overall, our metabolic results indicate that the anti-cancer effects of marjoram have an important impact on the utilization of energy sources by MCF-7 cells since they lead to increased utilization of alternative energy sources, although they are not capable of completely obliterating the difference between cancerous and control cells in the utilization of energy sources linked to anaerobic metabolism. Moreover, marjoram extracts reduce the metabolic response of cancerous cells to stimulant/catabolic compounds and steroid hormones.

As expected, exposure to doxorubicin was deleterious to both cancerous and control cells, dramatically reducing the production of NADH across all PM-M arrays, probably due to its high cytotoxicity. The difference in the metabolic response induced by doxorubicin as compared to baseline and marjoram extracts is evident from the graphics in Figure 6, where it is possible to appreciate that both MCF-7 and control cells produced no detectable NADH in the presence of doxorubicin.

## 4. Discussion

After data collection and analysis, the marjoram extracts exhibited the most positive outcomes of MTS tests in both MCF-7 and MCF-12A cell lines. When treated with these extracts, the MCF-7 cells displayed cytotoxic effects, while the MCF-12A cells displayed mitogenic effects (Figure 1, Figure 2, Figure 3, Figure 4 and Figure 5). Therefore, it can be concluded that marjoram extracts promote effective anti-cancer effects on MCF-7 cells while showing no adverse effects on normal breast cell lines. In fact, their mitogenic effects on MCF-12A cells may be beneficial during a treatment protocol.

Moreover, the assessment of the metabolic impact of candidate compounds confirms that the phytochemicals contained in the marjoram extracts are capable of partially correcting the Warburg effect in MCF-7 cells by reducing the efficacy of producing energy through anaerobic pathways and forcing the cancerous cells to recruit more alternative energy sources. This anti-glycolytic activity aligns with what has been described in an animal model [3], where extracts of *O. majorana* promoted a more efficient utilization of glucose-related energy sources via aerobic pathways. Since the Warburg effect and the related disrupted mitochondrial function are mechanisms common to many types of cancers [18], the potential impact of marjoram’s phytochemicals on the balance between aerobic and anaerobic metabolism may offer promising avenues for the utilization of these compounds in several chemotherapeutic protocols. It is plausible to hypothesize that the metabolic effects of marjoram may exert their main action on the utilization of glucose, as shown in studies exploring the effect of this herb in diabetic models [3], and the secondary effects of the increased efficacy of the utilization of high-level energy sources may be responsible for most of the other metabolic findings: for example, the lower levels of NADH generated by cancer cells in the presence of stimulant/catabolic hormones after exposure to marjoram may be related to the more efficient energetic production—via better utilization of glucose by aerobic pathways—that will not require further stimulation by such hormones.

The most remarkable results produced by marjoram extracts were observed in the presence of metabolic effectors. While at baseline, MCF-7 cells showed a significantly higher production of NADH than controls in response to stimulant/catabolic compounds, such as methylxanthine and epinephrine, and steroid hormones such as progesterone, estradiol, testosterone, and aldosterone. After exposing the cells to marjoram extracts, the energy production in cancerous cells was reduced without significant changes in control cells, leading to an inversion of the ratio between MCF-7 and the controls (Figure 6). These data suggest an important role of marjoram’s phytochemicals in cellular pathways regulating the response to key metabolic effectors and paving the way to future studies exploring further molecular targets for the therapeutic application of these compounds.

A recent study explored the anti-cancer properties of *O. majorana* using a similar approach (MTT assay) and the same cell line (MCF-7): the results are consistent with our findings and indicate a decreased cell proliferation and an increased induction of DNA damage [19]. However, the authors focused on the cells’ expression profile while we investigated the metabolic profile, providing complementary evidence to the proposed anti-cancer role of marjoram. Other studies produced evidence supporting a role either specifically against breast cancer [20] or other types of cancer [21,22], and overall underlined the efficacy of marjoram-derived active principles at disrupting cancerous cell growth and proliferation.

Our study presents several limitations, including the small number of cell lines analyzed, the utilization of a single concentration for the tested compounds, and the lack of validation using genomic and transcriptomic analysis to confirm the impact of marjoram extracts on specific metabolic pathways. However, the consistency of our findings with previous works supporting the anti-cancer effects of marjoram extracts [12,15,20], the utilization of two independent approaches, and the experiments’ design based on multiple technical replicates for both MTS and Biolog assays, provide us with confidence in the results of our pilot study: we generated promising evidence that the phytochemicals in extracts of *Origanum majorana* possess anti-cancer properties exerting cytotoxic effects on breast cancer cells mediated by the disruption of some of the metabolic alterations leading to cancer proliferation, while they produce no negative effect on cells from normal breast tissue. The findings of this preliminary study invite further functional assessments to validate the anti-cancer effects of marjoram in both in vitro and in vivo models, as well as in both primary and metastatic forms of cancers. In the continuous effort to pursue precision medicine and offer effective therapeutic protocols minimizing adverse effects, marjoram extracts are promising candidates that should be considered in future protocols for the treatment of breast cancer.

## Data Availability

All data generated or analyzed during this study are included in this published article and are available by request. Data is not publicly available due to privacy or ethical restrictions.

## Supplementary Materials

Table S1, TTEST of MCF-7 Control to Marjoram, Control to Doxorubicin, and Marjoram to Doxorubicin, listing the raw data of our metabolic profiling experiments can be downloaded at: https://www.mdpi.com/article/10.3390/metabo13101083/s1.
